# Diagnostic Clues in Pediatric Nutcracker Syndrome: From Two Clinical Cases to Current Literature Analysis

**DOI:** 10.3390/children9121988

**Published:** 2022-12-17

**Authors:** Speranza Cioffi, Federica Di Domenico, Giuseppina Russo, Angelica De Nigris, Stefano Guarino, Emanuele Miraglia del Giudice, Pierluigi Marzuillo, Anna Di Sessa

**Affiliations:** Department of Woman, Child and General and Specialized Surgery, University of Campania “Luigi Vanvitelli”, 80138 Naples, Italy

**Keywords:** nutcracker, syndrome, left, renal, vein, compression, diagnosis, treatment, children

## Abstract

Nutcracker syndrome (NCS) is a rare pediatric disease caused by left kidney vein compression. Besides the “Triade’s symptoms”, including hematuria, proteinuria, and flank pain, a wide spectrum of clinical manifestations has been reported. As the significant hemodynamic changes secondary to the dilatation of the left renal vein, serious consequences such as renal vein thrombosis and severe anemia might occur in these children. NCS diagnosis includes a variety of invasive and non-invasive imaging tools, but cutoff values need to be further validated. A conservative treatment represents the most common therapeutic approach for these patients, but operative options are available in selected cases. To complicate matters, a standard diagnostic and treatment algorithm is currently lacking and scientific pediatric evidence in this field is still poor and limited. In this perspective, early recognition of NCS is crucial but challenging for pediatricians. Therefore, a better knowledge of the disease is recommended. Starting from two different clinical presentations of NCS, we aimed to provide a comprehensive overview of the disease in children.

## 1. Introduction

Nutcracker syndrome (NCS) represents a rare disease in childhood [1,2] defined as a symptomatic compression of left renal vein (LRV) between mesenteric artery and aorta in anterior nutcracker (as the most common form) or between aorta and vertebral column in posterior nutcracker syndrome with a subsequent spectrum of clinical features [1,2,3]. The nutcracker phenomenon (NCP) is a consequence of the decrease in the angle formed between the aorta and the superior mesenteric artery compression LRV without any symptoms [1,4,5].

While NCS has been largely characterized in adults [1,4], pediatric data are still limited as the challenging diagnosis of the disease at this age [2,6,7].

Indeed, the wide variability of NCS in clinical presentation in children (ranging from hematuria and proteinuria to hypertension and nephrolithiasis) has made difficult a comprehensive overview in this field [8].

In an attempt to fill this gap, we aimed to provide a pediatric landscape of diagnostic work-up and treatment of NCS in children through the initial presentation of two different clinical cases.

## 2. Case Reports

### 2.1. Case 1

A 10-year-old girl came to our attention for occasional finding of proteinuria at urine dipstick. She presented with a three-month history of isolated proteinuria between 350 and 400 mg/24 h (normal value for her body surface area = 110 mg/24 h) and with a spot urine protein/creatinine ratio (UPr/UCr) between 0.7 and 0.9 mg/mg (normal value <0.2 mg/mg). Symptoms as edema, gross hematuria, urinary tract infections, rashes, fatigue, joint pain, and drug use (e.g., non-steroidal anti-inflammatory) were denied. Clinical examination was unremarkable. Her weight was 31 kg (10–25th percentile), height 145 cm (50–75th percentile), Body Mass Index (BMI) 14.8 kg/m^2^ (10th percentile), and blood pressure levels were normal. Serum creatinine and albumin levels were within the normal range (albumin = 4.2 g/dL, creatinine = 0.58 mg/dL). At our observation, the presence of this proteinuria degree at 24 h urine collection and at UPr/UCr on spot urine collected during the day was confirmed. Evaluating the urine dipstick and UPr/UCr on urine sample collected in supine position, the absence of proteinuria with UPr/Cr 0.17 mg/mg was found. These data were confirmed 4 months later.

**Figure 1:**
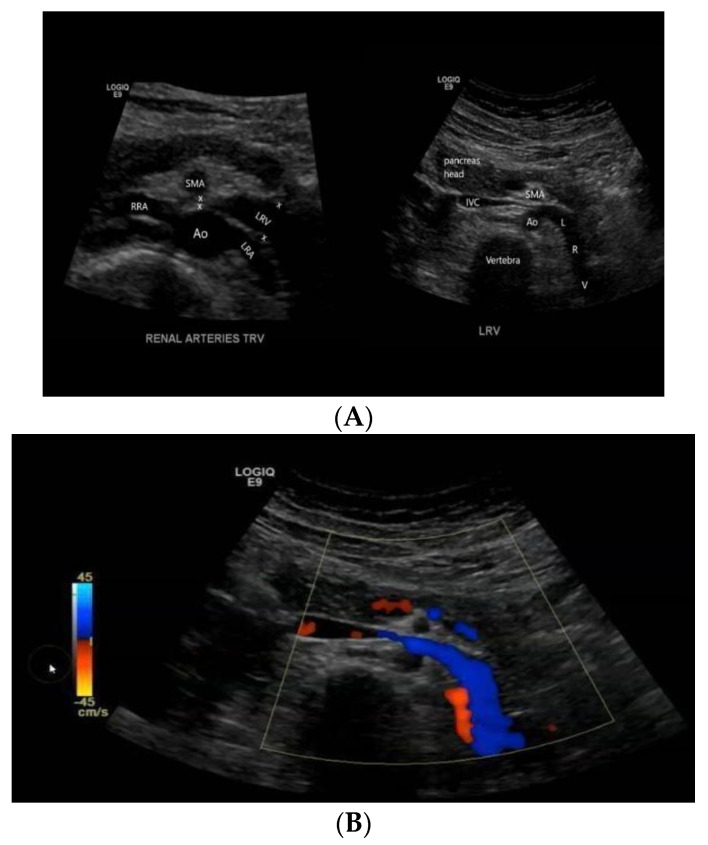
A transversal ultrasound section (**A**) showing the anatomical relationship between LRV, aorta, and SMA and the impingement of LVR, confirmed by Doppler imaging (**B**).

As orthostatic proteinuria might be attributable to NCS, the patient underwent kidney ultrasound with Doppler sonography (Figure 1). At renal ultrasound examination, both kidneys showed normal size, location, and echogenicity. More, Doppler renal ultrasound showed left renal vein compression, expressed by different diameter at the hilum (6 mm) and in its passage between the mesenteric artery and aorta (1.5 mm). The right renal vein diameter was of 4 mm at hilum. NCS was the final diagnosis. As the lack of significant impact of symptoms on the quality of life of the patient, she was eligible for a conservative approach and discharged home to continue three-to six months ambulatory follow-up with control examinations.

### 2.2. Case 2

A 13-year-old boy presented with 5 days of isolated macrohematuria during Epstein–Barr virus infection. His medical history was not contributive. A previous urine dipstick (at 10 years of age) was negative. At our observation, his anthropometric evaluation was as follows: weight 43.3 kg (10–25th percentile), height 160 cm (50th percentile), BMI 16.9 kg/m^2^ (25th percentile), and normal blood pressure levels. Physical examination was regular. Serum creatinine and complement proteins C3 and C4 were within the normal range. In the past, he sometimes complained of discomfort in both flanks (few seconds, twice a week). His dipstick showed Hb+++ with 10 red blood cells (5 dysmorphic, 2 acanthocytes, and 3 eumorphic) for high power field at urine microscopy. Proteinuria was absent both at 24 h urine collection (109 mg/24 h) and at UPr/Cr on spot urine (UPr/UCr 0.18 mg/mg). During the following 6 months, persistent micro-hematuria at urine dipstick (Hb++/+++) without proteinuria was detected. Therefore, a renal ultrasound with Doppler sonography was performed. Renal ultrasound was normal. At Doppler ultrasound, a reduction in the caliber of the left renal vein at the level of aorto-mesenteric compass (about 2 mm) with caliber of the upstream segment of about 7.5 mm was observed. In comparison, the caliber of the right renal vein was 6 mm accomplishing the diagnosis of NCS. Conservative management was chosen as the patient had minimal symptoms. He was scheduled for follow up visits every three to six months to evaluate blood pressure, complete blood count, and kidney function tests.

## 3. Clinical Features of NCS

**Figure 2:**
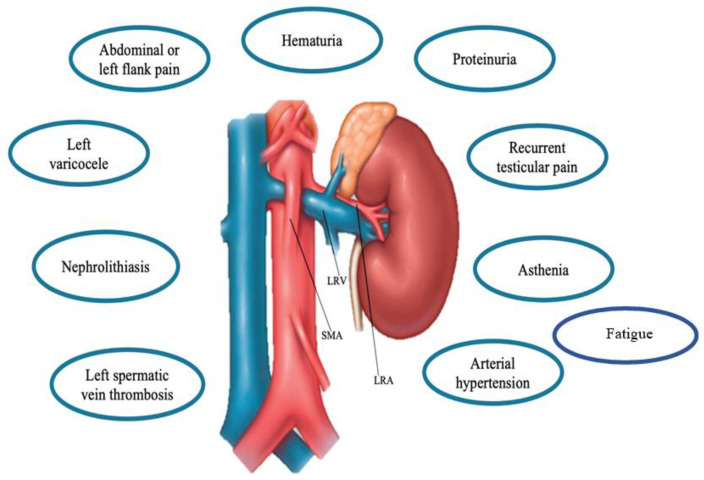
Main clinical features of the Nutcracker Syndrome. Abbreviations: LRV: Left renal vein, SMA: superior mesenteric artery, LRA, left renal artery.

Clinical presentation of NCS in children might widely vary as the hemodynamic consequences of anatomic changes [9]. In fact, LRV compression increases the retrograde venous pressure consequently affecting also other related vessels (e.g., left internal spermatic vein) resulting in different clinical manifestations [10]. Given its different degree of compression, different clinical features [2,5,11,12] ranging from asymptomatic cases to micro- and macrohematuria (intermittent or persistent), orthostatic proteinuria, renovascular hypertension, abdominal pain, (left-sided) flank pain, dysmenorrhea, testicular or scrotal pain, left varicocele, nephrolithiasis, calciuria, and fatigue may occur [6,8,13,14]. (Figure 2). In particular, proteinuria, macrohematuria, and flank pain have been reported as the most frequently encountered symptoms [2,5,8,9].

Although NCS might occur in patients of any age, a peak has been found in lean or underweight children aged 10–14 years, with a slight female predominance [10].

From a clinical point of view, two different NCS subtypes such as typical (or renal) presentation and atypical (or urologic) presentation have been described [1,2]. Typical clinical presentation includes hematuria (micro- to macrohematuria), and orthostatic proteinuria with or without flank pain. Conversely, abdominal pain, varicocele, dyspareunia, dysmenorrhea, fatigue, and orthostatic intolerance belong to the atypical presentation, rarely occur [15].

Due to the entrapment of LRV, orthostatic proteinuria (with or without microscopic hematuria) represents one of the most common symptoms in children with NCS with an estimated prevalence up to 75% [2,16]. In addition, abdominal or (left) flank pain also represents a major feature of the syndrome [1,2]. Based on the limited current data [2,15,17] the degree of proteinuria has been found to be variable and its incidence higher during puberty [15]. Moreover, no resolution of proteinuria overtime has been described in a percentage of these patients [14].

As the lack of robust evidence in this field, the mechanism of orthostatic proteinuria is still not fully elucidated. Changes of renal hemodynamic and elevated norepinephrine and angiotensin II levels have been supposed as potential pathogenic factors [15].

Hematuria (both macro- and microhematuria) represents another frequent clinical finding of NCS with an estimated prevalence up to 76% [2]. It is due to the rupture of thin-walled varicose veins and subsequent bleeding into the renal collecting system because of increased venous pressure [2,14]. Compared to adult patients, NCS children more frequently have microscopic hematuria without pain [6]. In anecdotic cases, NCS patients presenting with hematuria underwent an acute blood transfusion owing to intense anemia [6,15].

Atypical left flank pain occurs in one-third of pediatric patients with NCS and is usually explained as referred visceral pain secondary to LRV dilation [5,14]. Particularly, it is considered as a component of the well-known “Triade’s” symptoms of NCS including hematuria, proteinuria and flank pain [5].

An atypical diffuse abdominal pain encountered in 10% of the pediatric cases as a consequence of pelvic venous congestion [5,11,14,15]. Due to the inflammatory cascade activation triggered by venous hypertension, both flank and abdominal pain might occur in these patients [15].

Hypertension does not represent a classic sign of NCS and only a few reports have described NCS accompanied by hypertension in children. In case of secondary hypertension not linkable to other factors and in presence of a history of hypertension unresponsive to antihypertensive drugs, NCS should be considered as a potential cause. The underlying mechanism is complex and could be explained by an increased plasma renin activity and aldosterone levels in the peripheral blood despite the absence of renal artery stenosis or a renin-producing tumor [12].

Among uncommon NCS features, varicocele affects a small part of patients and usually occurs on the left side [10]. Its development has been related to high pressure in LRV and other vessels draining in the LRV such as left internal spermatic vein [10,15].

Moreover, chronic fatigue syndrome has been occasionally associated with NCS with high LRV-inferior vena cava pressure gradients [14]. Symptoms of autonomic dysfunction such as hypotension, syncope, and tachycardia could be observed but in rare cases [15].

The angle between the aorta and SMA is commonly between 38° and 65° [1,2], and space is enclosed by lymph nodes, mesenteric fat, and other soft tissues [1,2,5]. Some studies [2,5] have shown that the body fat content correlates with the fat content between the aorta and superior mesenteric artery and subsequently modulates the aforementioned angle [2,5]. In fact, the absence of intra-abdominal fat in patients with marked weight loss or leanness might promote the development of symptoms by narrowing the space between SMA and the aorta [5]. Instead, a considerable increase in BMI has been correlated with symptoms regression [2].

The clinical variability often might overlap with other clinical entities (e.g., Henoch–Schönlein purpura, IgA nephropathy, membranous nephropathy, and idiopathic hypercalciuria with nephrolithiasis), and the lack of standard diagnostic criteria further complicates the diagnostic work-up of NCS [14]. Of note, the relationship between the coexistence of NCS/NCP and glomerulopathy is still unclear [14,15,18]. To date, there is no consensus, but in patients diagnosed with LRV entrapment as well as in those with diagnosed glomerulonephritis and persistent hematuria even after treatment, a potential overlap should be considered [18]. Supine/standing urinalysis can help distinguishing NCS with or without glomerulopathy. Nonorthostatic urine abnormalities (e.g., proteinuria and/or hematuria) might be associated to glomerulopathy, as demonstrated in particular by the persistence of glomerular hematuria [19,20].

## 4. Diagnosis of NCS

Although a dilatation of LRV without clinical manifestations is frequently found in radiological images [2,21], the diagnosis of NCS can be demanding also in patients with highly suspicious clinical history as the current lack of standard diagnostic criteria [22,23,24]. The length of LVR is 6–10 cm. This vessel receives tributaries of different sites such as left adrenal, left gonadal, ureteral, and communicating lumbar veins [4]. Commonly, LRV diameter has an average of 4 to 5 mm, but some variations of normal anatomy should be considered prior NCS diagnosis or prognosis [4]. In addition, the noteworthy role of well-developed collaterals in underestimating NCS diagnosis should be highlighted [1,2,5].

When a thorough history and physical examination suggest NCS [25], urine analysis represents a first step to detect possible manifestations, such as repeated hematuria, either gross or microscopic; calciuria; and (orthostatic) proteinuria [2].

In this framework, extensive diagnostic procedures are required to confirm the diagnosis and the initial noninvasive work-up includes a Doppler ultrasound, computed tomography (CT) (with both arterial and portal venous phase), or a magnetic resonance imaging (MRI) using angiography sequences [26,27,28]. The main non-invasive findings are reported in Table 1.

First-line diagnostic options include abdominal ultrasound and doppler studies with a documented sensitivity and specificity of 82.3% and 89–100%, respectively [2,8].

For Doppler US, the application of ratio criterion can be challenging [24,33]. In fact, optimal cutoffs are of limited utility since the small LRV sampling area of children and the measurements variability positioning-related [2,15,34]. In particular, reference ratios might result slightly higher in the upright position likely due to gravity acting on bowel and mesentery, pulling the SMA downward and increasing LRV stenosis [22,35] as well as to the patient body habitus [36]. Moreover, US transducer compression as a tool to better demonstrate the LRV may cause additional compression of this vessel [26]. However, it represents an overall easy and feasible screening option especially in a pediatric context with the advantage of the lack of radiation and of invasiveness [36].

In selected and rare cases, invasive assessment might require a measurement of the pressure gradient between the LRV and inferior vena cava (IVC) by catheterization [27]. In the normal population, the pressure gradient between the LRV and IVC is less than 1 mm Hg [27]. When this pressure gradient exceeds 3 mm Hg, a definitive diagnosis of NCS can be made [27,37,38].

In addition to non-invasive tests (e.g., Doppler US, CT, or MRI) [8,22,39], phlebography is reserved for severe cases requiring an interventionist approach or for diagnosis confirmation through catheter venography and pressure measurement [2,25].

## 5. Treatment of NCS

As the lack of diagnostic consensus on Pediatric NCS, its management is still controversial. Commonly, the best approach to be used for each case should be determined by the severity of symptoms, the age of the patient, and the stage of the syndrome [1,2]. In particular, there is robust evidence that a conservative approach (e.g., “watch-and-wait” strategy) represents a valid first-line treatment for children diagnosed with NCS [8,40]. Indeed, a potential spontaneous resolution of NCS has been often observed in childhood as the development of adipose tissue development or of collaterals further improving LRV hypertension [5].

In this age group, during a wait and see monitoring of 24 months, instead of 6 months as for adults, a spontaneous and complete remission of symptoms has been demonstrated in 75% of cases [8,40]. Several possible underlying mechanisms have been hypothesized [1,2]. The growth process, with the physiological increase in retroperitoneal fat and fibrous tissue, may reduce the compression of the LRV by the aorto-mesenteric angle with a subsequent clinical improvement. In addition, avoiding any sporting activities potentially related to symptoms and correcting any posture disorders such as scoliosis have been considered as further therapeutic recommendations [2,8,14,40,41].

In case of persistent orthostatic proteinuria, a treatment with angiotensin converting enzyme inhibitors (ACE-i) has been proposed [42,43] with a considerable effectiveness [22,42].

Remarkably, rare and selected cases presented with severe abdominal or (left) flank pain, recurrent gross hematuria, renal dysfunction, left varicocele, anemia, and persistent symptoms (after 24 months of conservative treatment) might require a more complex and invasive management such as surgical and endovascular techniques [2,29,44]. LRV transposition, with or without renal autograft, represents the most preferred surgical technique [45]. LRV is excised from the IVC and reimplanted distal to the superior mesenteric artery (SMA) with demonstrated success in adults and good results in children [39,42,44].

Despite possible complications such as thrombosis, restenosis, retroperitoneal hematoma, and paralytic ileus, this technique has been associated with a long-term resolution of symptoms that exceed the aforementioned related risks [2,8,39,42,44].

Left renal autotransplantation might represent an additional invasive option for LRV pressure levels normalization [44] but further potential complications such as prolonged renal ischemia for anastomosis of renal arteries and ureters limit its usefulness [29,44,46].

Other possible techniques for NCS operative treatment include SMA transposition, nephropexy, nephrectomy, renocaval bypass, left gonadal vein transposition, or laparoscopic procedures (laparoscopic splenorenal venous bypass, and laparoscopic LRV-IVC transposition) [2,29,44]. An additional therapeutic option is represented by the endovascular approach with the implantation of a self-expanding stent in the LRV [2,29,44,47,48]. Although it represents the less invasive approach, it is not preferred due to the possible risks associated with stent migration and the difficult management of anticoagulant therapy post-stenting in children [29,44,47,48].

In view of the current data from the literature [1,2,25], a conservative treatment represents the pediatric first-line therapy, as supported by the high success rate observed in these patients [2,8,44].

As the delicate phase of childhood, other treatment options should be considered in selected cases after a careful risk-benefit evaluation to make the best therapeutic choice.

## 6. Conclusions

Owing to its limited knowledge in children, NCS represents a less-defined and often underdiagnosed disease with a large spectrum of potential symptoms. In this framework, both our cases confirmed and corroborated the wide clinical phenotypical variability (ranging from hematuria, proteinuria, and flank pain—as the most frequent symptoms–to varicocele, nephrolitiasis, and asthenia—as rare features) that might result in an easily missed diagnosis. NCS represents an uncommon cause of hematuria and proteinuria in children, but both features are the most common presentations of this syndrome. Therefore, a high index of suspicion and awareness of its clinical variability are required for pediatric nephrologists to avoid misdiagnosis or late diagnosis.

To complicate matters, the available data from the literature in this field arise from small pediatric case series, further preventing a large and validated knowledge of the disease. More, the lack of standard clinical criteria for NCS undermines the correct timing of the diagnosis.

Given the potential serious complications including renal vein thrombosis and severe anemia secondary to hematuria, increasing knowledge of NCS is crucial for its early identification. Although a conservative approach has been recognized as a first-line option, surgery might be a helpful approach in unsuccessful cases and persisting and/or severe symptoms, but several aspects limit its use in clinical practice.

Taken together, NCS diagnosis and treatment represent a great challenge for pediatricians. Therefore, a deeper understanding of the disease is needed to achieve an accurate and in time diagnosis and to improve the overall NCS management in children.

## Figures and Tables

**Table 1:** Main non-invasive findings in NCS.

Feature Description	References	Normal Values/Notes	Reference Values Compatible with NCS	Instrumental Technique
AMA Angle between the abdominal aorta and the SMA	[1,22,29,30,31]	The normal AMA is between 38° and 65°. A smaller AMA than this range of value, causes an entrapment of LRV with a significant increase in the pressure gradient between LRV and IVC	<35°	Doppler US, CT or MRI
Beck angle Angle between the aorta and SMA in the point of entrapment of LRV (at the aorto-mesenteric angle)	[1,4]	The beck sign represents the stenosis of the LRV at the aorto-mesenteric angle.	<32°	Doppler US, CT or MRI
LRV diameter ratio Comparison between the antero-posterior diameters of the LRV at the renal hilar and stenotic Aorto-mesenteric portion.	[1,4]	A LRV diameter ratio higher than the cut-off value is a specific sign for NCS and has a positive predictive value of 100%	>4.9	Doppler US, CT or MRI
PVR Peak velocity ratio across the entrapped LRV (between the renal hilum and aortomesenteric area	[1,26,27,32]	The best PV ratio cutoff values for the differentiation of the NCS was 4.7 in children.	>4.7	Doppler US

Abbreviations: AMA: Aorto-mesenteric angle; CT: computed tomography; IVC: Inferior Vena Cava; LRV: left renal vein, MRI: magnetic resonance imaging; PVR: peak velocity ratio, SMA: superior mesenteric artery US: ultrasound.
